# Supplementary material for: The interactome of intact mitochondria by cross-linking mass spectrometry provides evidence for coexisting respiratory supercomplexes
Source: Mol Cell Proteomics. 2017 Dec 8;17(2):216–32. doi: 10.1074/mcp.RA117.000470 (PMC5795388; doi:10.1074/mcp.RA117.000470)
Supplement: Supplemental Data [file supp_RA117.000470_133922_0_supp_23978_fzffwf.zip › spectra_annotation/mito_spectra_annotation/357-1-14-1-6-1.pdf]

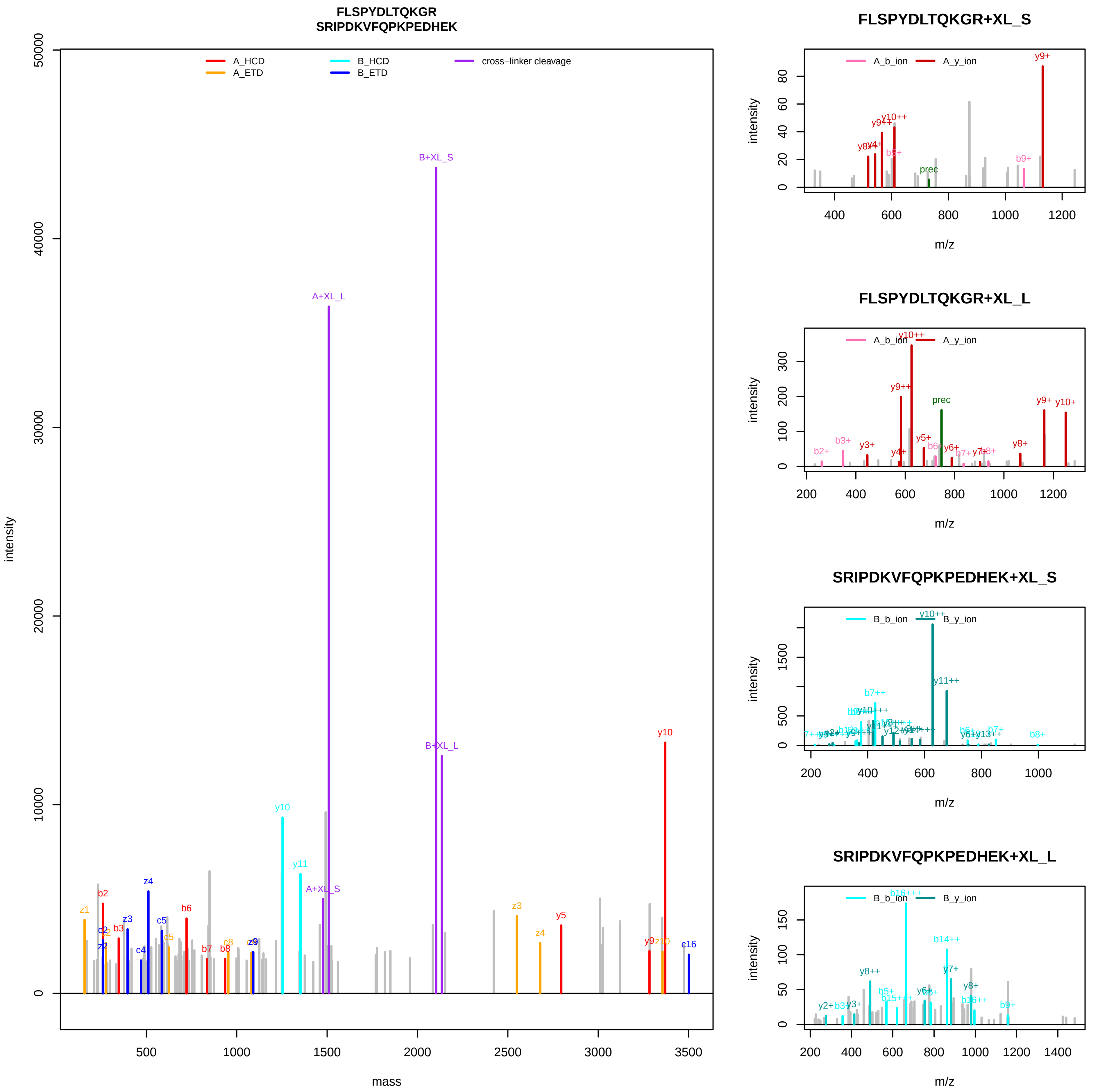

FLSPYDLTQKGR
SRIPDKVFQPKPEDHEK
A_HCD
A_ETD
B_HCD
B_ETD
cross−linker cleavage
intensity
mass
0
10000
20000
30000
40000
50000
500
1000
1500
2000
2500
3000
3500
B+XL_S
A+XL_L
B+XL_L
A+XL_S
y10
y11
y5
z3
z4
b2
b3
b6
b7
b8
z1
z3
z4
c4
c5
c8
c16
FLSPYDLTQKGR+XL_S
A_b_ion
A_y_ion
y9+
y10++
y9++
prec
b9+
m/z
FLSPYDLTQKGR+XL_L
y10++
y9++
prec
y9+
y10+
y5+
y6+
y3+
y8+
b2+
b3+
SRIPDKVFQPKPEDHEK+XL_S
B_b_ion
B_y_ion
y10++
y11++
b7++
b8+
SRIPDKVFQPKPEDHEK+XL_L
b16+++
b14++
y8++
y7+
y8+
b9+
y2+
y3+
